# Protecting Location Privacy for Outsourced Spatial Data in Cloud Storage

**DOI:** 10.1155/2014/108072

**Published:** 2014-07-06

**Authors:** Feng Tian, Xiaolin Gui, Jian An, Pan Yang, Jianqiang Zhao, Xuejun Zhang

**Affiliations:** ^1^School of Electronics and Information Engineering, Xi'an Jiaotong University, Xi'an 710049, China; ^2^Shaanxi Province Key Laboratory of Computer Network, Xi'an Jiaotong University, Xi'an 710049, China

## Abstract

As cloud computing services and location-aware devices are fully developed, a large amount of spatial data needs to be outsourced to the cloud storage provider, so the research on privacy protection for outsourced spatial data gets increasing attention from academia and industry. As a kind of spatial transformation method, Hilbert curve is widely used to protect the location privacy for spatial data. But sufficient security analysis for standard Hilbert curve (SHC) is seldom proceeded. In this paper, we propose an index modification method for SHC (SHC^∗^) and a density-based space filling curve (DSC) to improve the security of SHC; they can partially violate the distance-preserving property of SHC, so as to achieve better security. We formally define the *indistinguishability* and attack model for measuring the privacy disclosure risk of spatial transformation methods. The evaluation results indicate that SHC^∗^ and DSC are more secure than SHC, and DSC achieves the best index generation performance.

## 1. Introduction

The widespread use of location-aware devices promotes the development of various successful location-based services [1], and the amount of spatial information has grown at an exceptional speed over the past decade. This enormous spatial information should be maintained and processed by powerful data management system, which exceeds the capabilities of small business and individuals. Cloud computing adaptively allocates the resources and effectively reduces the manipulating and maintaining expenses for* data owner* (DO). Therefore, data outsourcing becomes a prevailing pattern and has earned widespread attentions from academia [2]. In this pattern, DO delegates the management of its data to a third-party* cloud storage provider* (SP), which maintains the data of DO and responses to the queries of* authorized user* (AU). However, as the data is outsourced to SP, DO cannot know where the data is stored and thus loses the direct control over the fate of their data. Therefore, protecting location privacy of outsourced spatial data is a big challenge with the development of spatial data outsourcing and location-based services [3].

In spatial data outsourcing pattern, spatial queries such as *K* nearest neighbor (*K*NN) or range queries are commonly issued by AU; in order to perform spatial queries, AU needs to share the location of the query point (query window) with SP [4]. However, the AU's location is highly sensitive information that, once compromised, can lead to various threats of privacy disclosure. For instance, malicious SP might sell commercially valuable information to the DO's rivals or can speculate the users' state of health based on the users' spatial queries.

For the privacy preserving of spatial data outsourcing, one simple solution is that DO first applies conventional encryption (e.g., AES) to the data locally and then outsources the encrypted data to SP. However, once the data is encrypted, traditional plaintext query techniques become invalid, and the AU cannot query or use the encrypted data effectively. This solution is inefficient for queries that only require a small fraction of the data. Applying computable encryption techniques [5, 6] will result in limitations when processing spatial queries. For example, if AU needs to find *K* nearest neighbor points of interest (POIs) to the query point *q*, although computable encryption techniques can calculate the encrypted Euclid distances between encrypted point *q* and each POI, it cannot sort these encrypted distances in ascending or descending order. Therefore, these encrypted distances should be sent back to AU who can decrypt them and find the top *K* results. By analyzing the process, we know that, in order to get correct query result, SP needs to compute the encrypted distances and send them back to AU, so the computation and communication complexity for SP is *O*(*n*), where *n* is the size of the outsourced dataset. As data explodes nowadays, this straightforward approach is not applicable in such scenario. Meanwhile, privacy information retrieval (PIR) [7] assures that no information about AU queries will be exposed to the untrusted SP; thus, it can achieve strong privacy-preserving level. But it will result in massive computation and communication cost and is not suitable for spatial data outsourcing.

To guarantee that DO and AU can query encrypted spatial data effectively while protecting the location privacy of outsourced spatial data, Hilbert curve is employed to transform the locations of both AU and POIs [8–12]. However, standard Hilbert curve (SHC) builds indexes of POIs using the same granularity in the spatial domain. If POIs densely distribute, its indexes generated by SHC will contain a lot of index values without the corresponding POIs; we call these values* null value segments*, which is easy for malicious SP to analyze and speculate the distribution of POIs in the transformed space. It will increase the location privacy disclosure risk of the outsourced spatial data. In this paper, we propose two index generation methods for outsourced spatial data and analyze the security and efficiency of these methods quantitatively. Our major contributions are summarized as follows.

1. We propose an index modification method for SHC (SHC^∗^) to improve its security, while a density-based space-filling curve (DSC) is also proposed for efficiency concerns.
2. A metric for measuring the privacy disclosure risk of the spatial transformation methods (e.g., SHC) is proposed, namely,* indistinguishability λ*. Additionally, we formally define an attack model for these spatial transformation methods; this model quantifies the attacker's background knowledge and applies a modified general attack method to reconstruct the original spatial dataset.
3. The evaluation results conducted on real-world datasets show that our spatial transformation methods can achieve better security than SHC.

The remainder of this paper is organized as follows.  Section 2 reviews related work.  Section 3 proposes our spatial transformation methods.  Section 4 presents the* indistinguishability* and attack model for security analysis. An empirical evaluation is presented in Section 5.  Section 6 concludes and discusses future research directions.

## 2. Related Work

### 2.1. Spatial Query Privacy Protection

Confidentiality has been addressed in the context of spatial queries. Mobile users issue spatial queries (e.g., range or* K*NN queries), which are answered by the service provider (e.g., Google Maps). Users do not want to reveal their exact location to the SP. In order to protect the spatial query privacy, the user location should be first generalized by a trusted location anonymizer [13], which processes the query and generates the anonymizing spatial region instead of the exact user location. Much of the work regarding spatial query privacy protection derives from *k*-anonymity model which is first proposed by Sweeny in database [14]. Gruteser and Grunwald [15] first proposed location *k*-anonymity on the basis of this model to protect the spatial query privacy. Based on location *k*-anonymity, Gedik and Liu [16] proposed a scalable architecture which supports location *k*-anonymity for a wide range of mobile clients with context-sensitive privacy requirements. Bamba et al. [17] proposed a framework for supporting anonymous location-based queries, which divides the geographical area of interest into grid cells and achieves *k*-anonymity by the grid.

Taking the anonymization policy into consideration, Deutsch et al. [18] proposed policy-aware location *k*-anonymity, which could defend against more realistic policy-aware attackers. As most of the location *k*-anonymity algorithms cannot effectively prevent location-dependent attacks when users' locations are continuously updated, Pan et al. [19] proposed an incremental clique-based cloaking algorithm to solve this problem. In addition to the location *k*-anonymity, a number of other query privacy-preserving methods were proposed. For example, Beresford and Stajano [20] introduced the mix zone to map the problem of spatial query privacy onto that of anonymous communication. By building mix zones at road network, Palanisamy and Liu [21] present a framework to protect location privacy of mobile users travelling on road networks.

The spatial query privacy-preserving methods hide the query users' location from the SP but do not protect the data being queried. Therefore, it is orthogonal to our problem.

### 2.2. Spatial Data Outsourcing Privacy Protection

Hacigümüs et al. [22] first introduced the idea of outsourcing database services to a third-party service provider. Then, they provided a solution for outsourced data privacy protection, by constructing index based on encrypted data and additional bucketing information, to support encrypted data query [23]. Later, aiming at one-dimensional numeric values, Agrawal et al. [24] have proposed an order-preserving encryption scheme (OPES) which supports efficient processing of queries at the SP. Afterwards, Huang et al. [25] have presented an outsourced data privacy-preserving approach which supports fuzzy query of encrypted string. All the above approaches are suitable for string or one-dimensional numeric values and they cannot be directly applied to privacy protection of spatial data.

Wong et al. [26] have studied* K*NN computation of encrypted tuples stored at untrusted SP and propose a method supporting SP to calculate the relative distance between two encrypted data points. As virtual dimension has been introduced, this method cannot build up index of encrypted data points efficiently. Therefore, when dealing with query request, SP needs to traverse all encrypted data points, which leads to relatively poor query efficiency.

In order to support query services on outsourced private spatial data, some researchers have employed space filling curves to transform POIs, which can support range and* K*NN query in the transformed space. In practice, SHC is applied in most circumstances. By using SHC, Ni et al. [8] have reduced extra work load due to setting users' parameters. Meanwhile, the computation and communication cost of range query has been reduced since the clustering and distance-preserving properties of SHC are superior. Khoshgozaran et al. [9] use SHC to map data points and query request to Hilbert transformed space and enable query services on encrypted spatial data. In addition, they introduce dual Hilbert curve technique, which improves the accuracy and efficiency of query. Ku et al. [10] study the query integrity assurance scheme. Based on SHC, the data points are encrypted with a symmetric key and indexed by Hilbert value. The original database and a sample of it are encrypted with different space encryption keys. Then the two encrypted datasets are combined and stored at SP for integrity verification.

According to the current research, SHC does not take the distribution of POIs into consideration when transforming the original space. In fact, it divides the space using the same granularity and generates Hilbert values so as to construct the indexes of POIs. In order to guarantee the security and query efficiency, a large enough curve order is needed to make sure that there are not two POIs with the same Hilbert value. Khoshgozaran et al. [9] pointed out that without the knowledge of Hilbert curve parameters, malicious SP can still find out the dense area of POIs in the transformed space by analyzing the number of POIs with the same or similar Hilbert values, which increases the location privacy disclosure risk of outsourced spatial data.

## 3. Spatial Transformation Methods

In order to protect the location privacy of spatial data, we need to transform the original locations of POIs. An ideal space transformation method should be a one-way function, which is easy to compute but difficult to invert. Meanwhile, to maintain the query efficiency of encrypted spatial data, the space transformation method should respect the spatial proximity of the original space. In this section, we first introduce the standard Hilbert curve, which is a representative spatial transformation method. Then, we propose two improved spatial transformation methods, which achieve better security than SHC.

### 3.1. Standard Hilbert Curve (SHC)

Space filling curves [27] have the above features and can be applied in spatial data transformation to protect location privacy for outsourced spatial data. Space filling curve passes through every partition of a closed space and has no intersection with itself. In this way, each point in multidimensional space will be mapped as a value to one-dimensional space. *Z* curve [28], Gray curve [29], and Hilbert curve are all space filling curves, which can be used for space transformation.

Compared to *Z* curve and Gray curve, Hilbert curve is widely applied due to its superior clustering and distance-preserving properties [11, 28–30, 34]. Similar to [11], we use *H*_*d*_^*N*^ to denote Hilbert curve with order *N* in *d*-dimensional space, where *N* ≥ 1 and *d* ≥ 2. In this way, *d*-dimensional integer space [0,2^*N*^−1]^*d*^ can be mapped to a one-dimensional integer set [0, 2^*Nd*^ − 1], which means that, for any POI *p* in *d*-dimensional space, there is a function *f* satisfying *V*_*H*_ = *f*(*p*), where *V*_*H*_ ∈ [0, 2^*Nd*^ − 1]. As one partitioned region may contain multiple POIs, different POIs may have the same index value for a given Hilbert curve.

**Figure 1:**
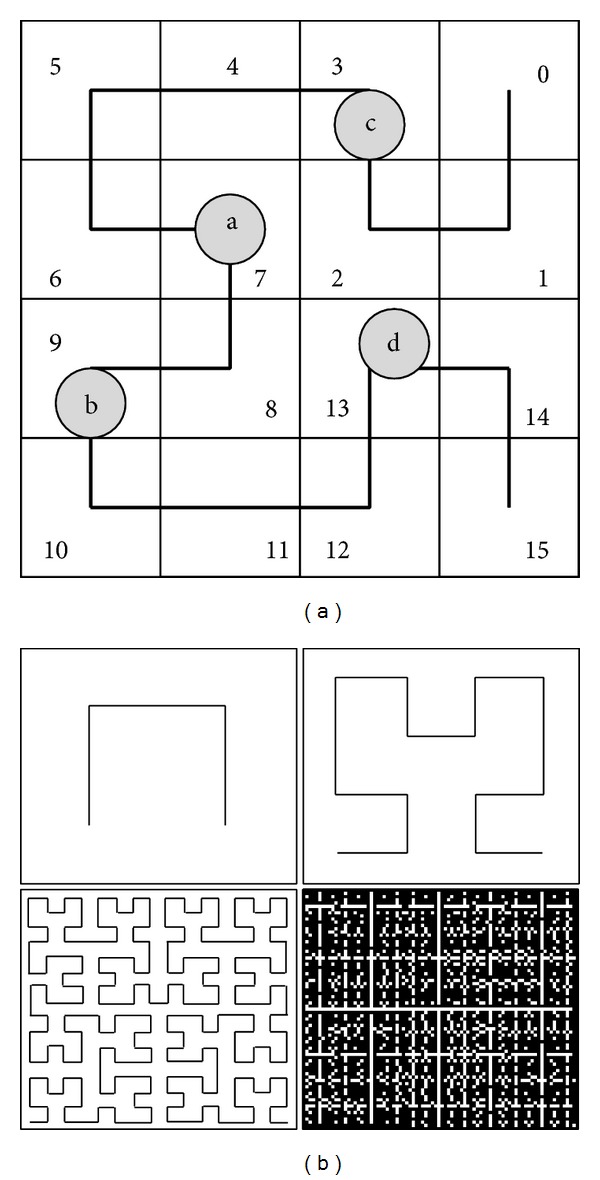
(a) A *H*_2_^2^ pass of the 2D space and (b) Hilbert curves with various orders.

Since the main goal of this paper is to protect the location privacy of POIs, we focus on the spatial transformations in two-dimensional space.  Figure 1(a) illustrates the process of transforming a two-dimensional space into Hilbert values. All POIs are traversed by a second order Hilbert curve and are indexed based on the sequence they are visited by the curve. So, in this example the POIs *a*, *b*, *c*, and *d* are represented by their Hilbert values 7, 9, 3, and 13, respectively.  Figure 1(b) depicts Hilbert curve with order 1, 2, 4, and 6. From the figure we can see that the larger the order is, the more fine-grained the Hilbert curve is.

### 3.2. Index Modification Method for SHC (SHC^∗^)

As real-world spatial datasets show aggregate distribution, the POIs' indexes generated by SHC contain many* null value segments*, which increase the privacy disclosure risk. Malicious SP has the whole indexes and may passively obtain some mappings between the POIs and indexes. By scanning the indexes, the attacker can easily find the continuous segments and* null value segments*. He can focus on this information and apply his background knowledge to estimate the location of unknown POIs.

**Figure 2:**
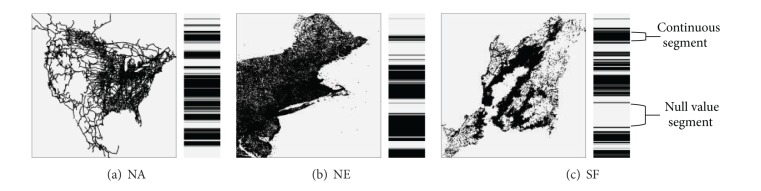
Visualization of POIs' indexes.

We study the distribution characteristics of the indexes generated by SHC. And the distribution of the indexes is depicted in Figure 2.

In Figure 2, the left picture represents the original dataset and the corresponding histogram represents the visualization of POIs' indexes. In histogram, the black lines represent the continuous segments and the gray parts denote the* null value segments*. If we compress these gray parts, the distribution of the indexes will become equilibrium. So it will be more difficult for the attackers to analyze, and the privacy disclosure risk will decrease. Based on this concept, we present an index modification algorithm to improve the security of SHC, denoted as SHC^∗^.

In Algorithm 1, based on the POIs' indexes *I*′ generated by SHC and user parameter max gap *M*, which denotes the max gap value between two indexes in the modified POIs' indexes I*, we compare the difference between two neighbor indexes; if the difference is beyond the max gap *M*, then the latter index should be modified using the forward index and *M*. The modified POIs' indexes I* should be updated with the new tuple, which contains the index *i* and encrypted POI. The time complexity of this algorithm is *O*(|*I*′|), where |*I*′| represents the cardinality of *I*′.

After applying Algorithm 1 to modify the POIs' indexes, the length of gray parts in Figure 2 will decrease dramatically, and the outsourced spatial datasets are more difficult for the malicious SP to attack.

### 3.3. Density-Based Space Filling Curve (DSC)

As SHC^∗^ starts executing after the indexes have been generated by SHC, SHC^∗^ takes more time to generate the POIs' indexes. Inspired by the idea that SHC can partition and transform the original space, we propose a density-based space filling curve (DSC), which takes the distribution of POIs into consideration. DSC partitions the spatial domain according to the* capacity*, which is the maximum number of POIs a partitioned region contains, denoted as *C*. It uses fractal rules of Hilbert curve to determine the visiting sequence of each partitioned region.

Two steps are involved in the DSC generation. (1) According to the* capacity*, the spatial domain is partitioned by quad tree structure [31]. And the generated partitioned regions will be represented as quad tree nodes. (2) Based on the curve orientation, starting point, and scaling factor given by DO, each partitioned region is traversed sequentially in accordance with the fractal rules of Hilbert curve, then the sequence number of each partitioned region is generated, and we call this number DSC value, which is used to build indexes of POIs.

**Figure 3:**
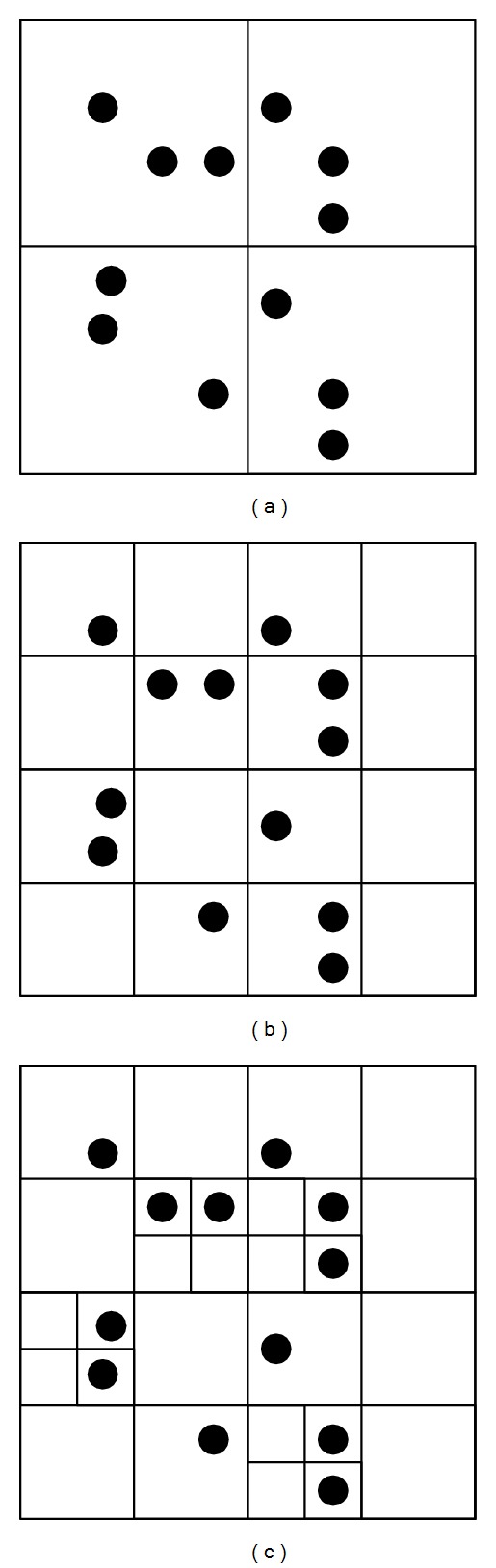
The quad tree partition: (a) *C* = 3, (b) *C* = 2, and (c) *C* = 1.

*Quad Tree-Based Space Partition*. In DSC, the spatial domain is partitioned by quad tree structure. The granularity is decided by* capacity C*, which means that, under current partition, if the number of POIs in region *R* exceeds *C*, we should continue to partition *R*, until the number of POIs in each partitioned region does not exceed *C*.  Figure 3 shows the different partitions using various *C*. From Figure 3 we can see that the smaller the *C* is, the more fine-grained the partition is.

DSC partitions the space and generates quad tree nodes corresponding to partitioned regions on the basis of POI dataset and* capacity*.

**Figure 4:**
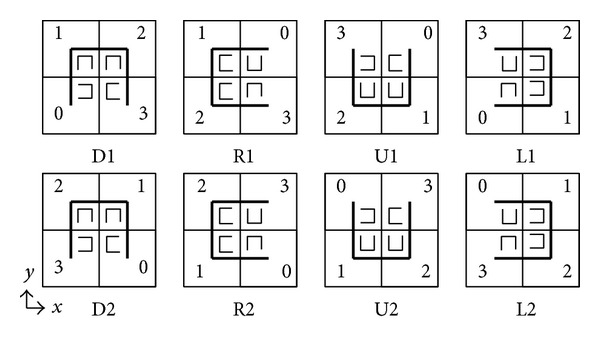
Fractal rules of Hilbert curve.

*Index Generation for DSC.* After partitioning spatial domain and generating quad tree nodes, it is necessary to generate the leaf nodes' DSC value and intermediate nodes' subcurve orientation and starting point according to the preset curve orientation and starting point of DSC, shown in Algorithm 2. Meanwhile, the index value of each POI is set the same as the DSC value of the partitioned region that the POI belongs to. This algorithm employs Hilbert curve fractal rules shown in Figure 4.

There are eight types of Hilbert curve fractal rules. Sequence number of subregion is assigned by the number in the quad. According to the rules in Figure 4, each subregion can be further partitioned to generate curves with higher order.

In Algorithm 2, *Q*_*O*_ is the subcurve orientation of node *Q*, *Q*_*S*_ is the subcurve starting point of node *Q*, *S* is a stack which stores quad tree nodes, *Z*_*D*_ is the DSC value of node *Z*, and *Z*(*i*) is the *i*th child node of *Z*. Algorithm 2 depth-first traverses quad tree *Q* according to preset curve orientation *θ* and starting point *S*_0_ and generates index value of each partitioned region based on the visiting sequence of leaf nodes. From the root node, we set the orientation and starting point of each intermediate node level by level according to the fractal rules in Figure 4 (lines 6–9). *T*_*O*_(*Z*_*O*_, *Z*_*S*_, *i*) and *T*_*S*_(*Z*_*O*_, *Z*_*S*_, *i*) represent the subcurve orientation and starting point of node *Z*'s *i*th child node, respectively.

Assuming the size of POI dataset is *n*. On average, Algorithm 2 needs to visit ∑_*i*=0_^log⁡_4_⁡(*n*/*C*)^4^*i*^ = (4*n* − *C*)/3*C* nodes, and each node will be visited only once. So the complexity of Algorithm 2 is *O*(*n*).

## 4. Security Analysis

In this section, we first analyze the security of SHC and propose* indistinguishability* for measuring the privacy disclosure risk of the space filling curves. Then we formally define the attack model, which quantifies the attacker's background knowledge and measures the security of space filling curves.

### 4.1. Privacy Disclosure Risk

As we know, malicious SP can identify the dense area of POIs in the transformed space by analyzing the number of POIs with similar or the same index values. In order to prevent multiple POIs from allocating the same index value, we need to increase the curve order of SHC. In practice, POIs densely distribute in most cases, and applying SHC to generate indexes of POIs may introduce many* null value segments*. In this way, malicious SP can analyze these* null value segments* and then find out the dense areas in the transformed space. Although using dummy values to reduce the* null value segments* [9] can explicitly eliminate these segments, by analyzing the query log, malicious SP can still discover the index values with low query frequency and then confirm the dummy values. In order to quantify the privacy disclosure risk, we employ the idea of information entropy and define* indistinguishability λ* as follows:

(1) $$\begin{array}{c} \lambda =-\overset{G}{\underset{i=1}{\sum }}\frac{\delta_{i}}{T}\log_{2}\frac{\delta_{i}}{T}-\overset{L}{\underset{j=1}{\sum }}\frac{\varphi_{j}}{T}\log_{2}\frac{\varphi_{j}}{T}, \end{array}$$

where *δ*_*i*_ represents the length of a* null value segment* in the POIs' indexes; *G* represents the number of* null value segments*; *φ*_*j*_ represents the length of a continuous segment in the POIs' indexes; *L* represents the number of continuous segments; *T* represents the total length of the POIs' indexes.* Indistinguishability* measures how* null value segments* and continuous segments affect the privacy disclosure risk. It is difficult for malicious SP to analyze the uniformly distributed indexes. According to the characteristics of information entropy, we learn that the more unevenly the* null value segments* and continuous segments distribute, the lower the* indistinguishability* is, and thus the higher the privacy disclosure risk is.

### 4.2. Attack Model

Because malicious SP may analyze the encrypted spatial data to estimate the original locations, DO wants to know the security level of spatial transformation methods. The malicious SP may obtain some background knowledge, for example, a subset of mappings between the original dataset *P* and transformed dataset *P*′. Then, he can employ some attack methods to estimate the original locations of the objects in *P*′ and get the estimated dataset P*. We use* estimation distortion DT*(*P*, P*) [4] to measure the average error between the original dataset *P* and the estimated dataset P* as follows:

(2) $$\begin{array}{c} DT\left(P,P^{*}\right)={\text{AVG}}_{p\cdot id=p^{*}\cdot id,p\in P,p^{*}\in P^{*}}||p-p^{*}||, \end{array}$$

where ||*p* − p*|| represents the Euclidean distance between *p* and p*. Obviously, a high *DT*(*P*, P*) value means that the spatial transformation method is difficult to attack. The estimation distortion *DT*(*P*, P*) could be used to measure the security level of a spatial transformation method.

We assume the attacker may obtain prior background knowledge of the outsourced spatial dataset. For example, an attacker may know some mappings between original locations and corresponding indexes. Due to the superior clustering and distance-preserving properties of SHC, the attacker can estimate the original locations of the indexes by the known mappings. For the sake of discussion, we assume that the attacker passively knows the following information.

1. A subset *A* ⊂ *P* of *n* POIs, *A* = {*a*_1_, *a*_2_, …, *a*_*n*_}, where *a*_*i*_ is the location of POI.
2. The corresponding subset *A*′ ⊂ *I*′ of POIs' indexes, *A*′ = {*a*_1_′, *a*_2_′, …, *a*_*n*_′}, where *a*_*i*_′ is the index of location *a*_*i*_.

As the attacker can only obtain *A* and *A*′ in a passive manner, he cannot actively choose them as he wishes. Although the attacker knows the POIs' indexes in *I*′ − *A*′, he does not know the location of any POI in *P* − *A*. So the attacker tries to estimate the original location of each index in *I*′ − *A*′. The spatial transformation method should make this estimation with a high *DT*(*P*, P*) value.

Yiu et al. [4] define the general attack model and propose a heuristic-based general attack method that allows the attacker to estimate a reasonable approximation of the original location, by exploiting his limited knowledge of the known mappings. Similarly, for an index *b*′ ∈ *I*′ − *A*′, we define its feature vector over *A*′ as follows:

(3) $$\begin{array}{c} V\left(b^{\prime },A^{\prime }\right)=\left(\left|b^{\prime }-a_{1}^{\prime }\right|,\left|b^{\prime }-a_{2}^{\prime }\right|,\ldots ,\left|b^{\prime }-a_{n}^{\prime }\right|\right), \end{array}$$

where |*b*′ − *a*_*i*_′| represents the absolute difference between *b*′ and *a*_*i*_′. As we know, there are* null value segments* in the POIs' indexes *I*′, and the clustering and distance-preserving performance of SHC is superior. Thus, the attacker should not compute the absolute difference for each index *a*_*i*_′ ∈ *A*′. For index *b*′, the attacker will only compute the absolute difference for the indexes close to *b*′ and these indexes should be continuous. These indexes constitute a subset of *A*′, denoted as *B*′, which varies with *b*′. So the feature vector of index *b*′ ∈ *I*′ − *A*′ should be revised as follows:

(4) $$\begin{array}{c} V\left(b^{\prime },B^{\prime }\right)=\left(\left|b^{\prime }-a_{1}^{\prime }\right|,\left|b^{\prime }-a_{2}^{\prime }\right|,\ldots ,\left|b^{\prime }-a_{m}^{\prime }\right|\right), \end{array}$$

where *B*′ = {*a*_1_′, *a*_2_′, …, *a*_*m*_′} represents a subset of *A*′, and *m* < *n*. For a location *p* in the original spatial domain, its feature vector is

(5) $$\begin{array}{c} V\left(p,B\right)=\left(||p-a_{1}||,||p-a_{2}||,\ldots ,||p-a_{m}||\right), \end{array}$$

where *B* = {*a*_1_, *a*_2_, …, *a*_*m*_} represents a subset of *A*, and this subset is corresponding with {*a*_1_′, *a*_2_′, …, *a*_*m*_′}. By choosing the candidate location *l* and then comparing the coherent pattern of feature vectors **V**(*b*′, *B*′) and **V**(*l*, *B*), the attacker can identify the original location of index *b*′ with low estimation error. For the direct comparison, the feature vectors should be normalized by their magnitudes, and we use* dissimilarity* [4] to measure the difference between *l* and *b*′ as follows:

(6) $$\begin{array}{c} \eta \left(l,b^{\prime }\right)=L_{1}\left(\frac{V\left(b^{\prime },B^{\prime }\right)}{\left|V\left(b^{\prime },B^{\prime }\right)\right|},\frac{V\left(l,B\right)}{\left|V\left(l,B\right)\right|}\right), \end{array}$$

where *L*_1_ is the Manhattan distance. So the attacker can estimate the original location of index *b*′ as l*, which is the candidate location *l* with the smallest* dissimilarity* value,

(7) $$\begin{array}{c} l^{*}=\underset{l}{\arg\;\min}\;\eta \left(l,b^{\prime }\right). \end{array}$$

Obviously, the computation of l* results in an infinite number of candidate locations in the original spatial domain. We assume the attacker employs a randomized numerical method to obtain an approximation of l* within acceptable time. By estimating each index *b*′ ∈ *I*′ − *A*′, the attacker can obtain an estimated dataset P*,

(8) $$\begin{array}{c} P^{*}=\left\{\underset{l}{\arg\;\min}\;\eta \left(l,b^{\prime }\right)\;|\;b^{\prime }\in I^{\prime }\right\}. \end{array}$$

In Section 5, we empirically study the security level of SHC, SHC^∗^, and DSC by applying the above modified general attack model.

## 5. Evaluation Results

We evaluate the performance and security of SHC, SHC^∗^, and DSC using four real spatial datasets [32]: North East USA (NE: 123, 593 POIs), San Joaquin County (TG: 18, 263 POIs), San Francisco (SF: 174, 956 POIs), and North America (NA: 175, 813 POIs). These datasets match well the private spatial data outsourcing mentioned in Section 1. The domain of each dataset is normalized to the unit square [0,1]^2^ and the experiments are carried out on Intel i5-2400 3.1 Ghz with 8 GB RAM. The start point, curve orientation, and curve scale factor are preset to (0, 0), D1, and 1, respectively.

### 5.1. Parameter Selection

When applying SHC or DSC to transform the spatial domain, each partitioned region will be allocated with a value. POI is assigned the same value as the partitioned region that it belongs to, and this value can be employed to build index of the POI set. When two POIs are in the same partitioned region, they will be assigned the same value. Let *α* represent the average number of POIs with the same index value; using SHC and DSC for indexing POIs, we measure *α* for each value of *N* and *C* in all datasets. Ideally we want *α* to be infinitely close to 1, meaning that there are not two POIs with the same index value.

**Figure 5:**
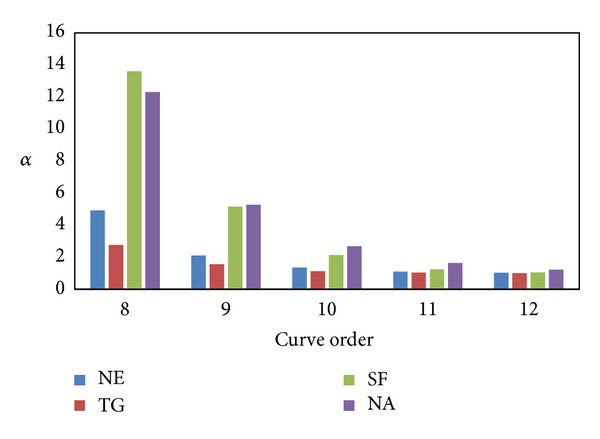
Average number of POIs versus curve order.

**Figure 6:**
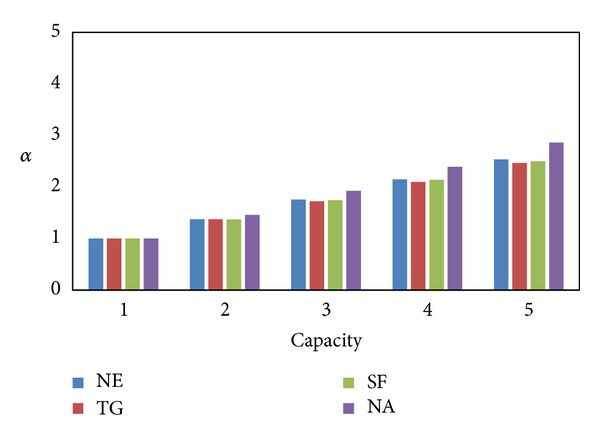
Average number of POIs versus capacity.

SHC^∗^ has the same *α* as SHC, so we only study the relationship between curve parameters and *α* for SHC and DSC to guarantee that the following experiments are conducted under the same conditions.  Figure 5 shows the *α* of SHC for different curve order *N*. The *α* of SHC drops drastically as curve order increases. When *N* = 12, *α* approaches 1 in NE, TG, and SF, but NA needs *N* = 13.  Figure 6 depicts the *α* of DSC for different* capacity C*. The *α* of DSC rises slowly as capacity increases. When *C* = 1, *α* = 1. So we apply this setting to SHC (SHC^∗^ applies the same setting as SHC) and DSC in the remaining experiments.

As a user parameter, the max gap *M* for SHC^∗^ could take any value. We study the relationship between* indistinguishability* and *M* for SHC^∗^ and find out that the* indistinguishability* of SHC^∗^ remains steady when *M* ∈ [5, 55] and drops when *M* > 60. For the sake of discussion in this paper, we set *M* = 10 in the following experiments, which effectively compress the* null value segments* and reduce the computation overhead.

### 5.2. Index Generation Comparison

The computation cost of index generation is an important metric for evaluating the spatial transformation methods. We set *α* = 1 for SHC, SHC^∗^, and DSC and compare their efficiency of generating index in different datasets. EDHO [33] and BIA [34] are index generation algorithms for SHC, while IGD proposed in this paper is the index generation algorithm for DSC. Because SHC^∗^ starts executing after EDHO or BIA, we use SHC^∗^-E and SHC^∗^-B to denote the different sequences of execution, respectively. The experiment is performed for 100 times and the index generation time (ms) is averaged.

Table 1 shows the index generation time of SHC, SHC^∗^, and DSC over different datasets. BIA generates index faster than EDHO, and SHC^∗^-B is faster than SHC^∗^-E, but they are all slower than IGD. Because SHC^∗^-E is consist of EDHO and SHC^∗^, it takes a little more time than EDHO. And the explanation for the result of SHC^∗^-B is similar with SHC^∗^-E. On average, the time cost by IGD is only 49.2%, 64.5% of that by EDHO and BIA, respectively. Sine DSC takes the distribution of POI dataset into consideration and chooses different curve orders in regions with different density, which enables sparse POI regions to use relatively low curve order and thus improves the computation efficiency.

### 5.3. Indistinguishability Comparison

We apply the parameter setting in Section 5.1 and calculate the* indistinguishability* of these spatial transformation methods when building POIs' indexes. Because only index values corresponding to POIs are used as POIs' indexes, which are continuous segments, we could sequentially scan the POIs' indexes and find out the gaps between the continuous segments, and these gaps are* null value segments*.  Table 2 shows the* indistinguishability* of SHC, SHC^∗^, and DSC for different datasets.

The* indistinguishability* of SHC^∗^ and DSC is significantly higher than that of SHC in all datasets. On average, SHC^∗^ and DSC are 68.4% and 64.9% higher than SHC, respectively. It implies that SHC^∗^ and DSC can maintain a very low privacy disclosure risk. By analyzing the characteristics of these spatial transformation methods, we can learn that, when SHC builds index on real spatial dataset, there will be a large amount of* null value segments*, and the average length of the continuous segments is quite different from that of* null value segments*. While DSC partitions the spatial domain according to the density, the number and length of* null value segments* will be far less than those of SHC, and the* null value segments* and continuous segments distribute equilibrium, making* indistinguishability* of DSC greatly larger than that of SHC.

**Figure 7:**
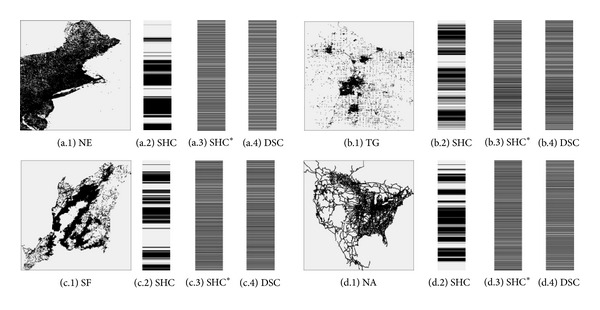
Visualization of POIs' indexes built by SHC, SHC^∗^, and DSC.

In order to compare the distributions of* null value segments* and continuous segments when employing different spatial transformation methods, we illustrate the POIs' indexes built by SHC, SHC^∗^, and DSC in Figure 7, where (a.1), (b.1), (c.1), and (d.1) denote the NE, TG, SF, and NA datasets, respectively. And 2, 3, and 4 represent POIs' indexes built by SHC, SHC^∗^, and DSC, respectively. As described in Section 3.2, the black lines of the histogram represent the continuous segments and the gray parts denote the* null value segments*. We can see that the length of the gray parts decreases dramatically after applying Algorithm 1 to modify the POI's indexes built by SHC. Because SHC^∗^ compresses* null value segments*, the total length of* null value segments* is far less than SHC, and the distribution of* null value segments* and continuous segments is much more equilibrium than SHC, which makes* indistinguishability *of SHC^∗^ much larger than that of SHC, leading to lower privacy disclosure risk. From this figure, we also find out that the distributions of the POIs' indexes built by SHC^∗^ and DSC are similar, and that is why the* indistinguishability *of SHC^∗^ is similar to that of DSC.

### 5.4. Attacks against SHC, SHC^∗^, and DSC

We apply the parameter setting in Section 5.1 and set the ratio of known set *A* to 1% based on the assumption that the attackers obtain limited background knowledge and the number of neighbor reference POIs to 15 which is the best value for POI estimation in our experiments. The attack method described in Section 4 is applied to reconstruct the original datasets.

**Figure 8:**
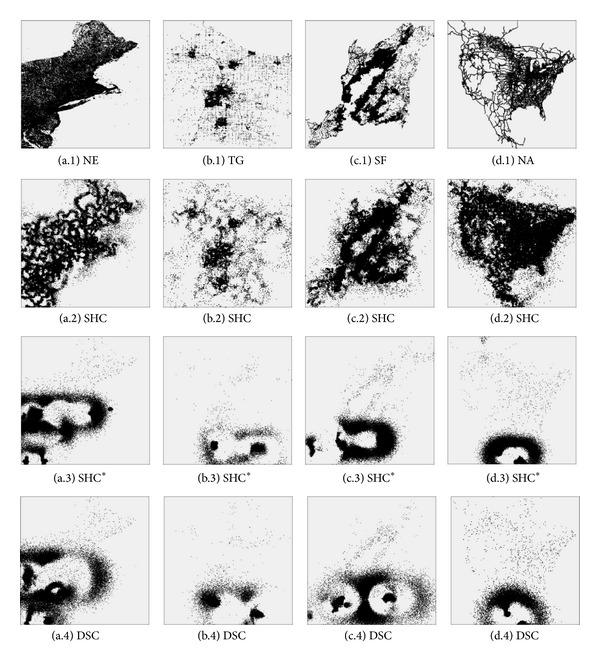
Visualization of attacks.

Figure 8 depicts the estimated datasets reconstructed by the attack method over the POIs' indexes generated by SHC, SHC^∗^, and DSC, where a, b, c, and d denote the NE, TG, SF, and NA datasets, respectively, while 1, 2, 3, and 4 represent the original datasets, the estimated datasets over SHC, the estimated datasets over SHC^∗^, and the estimated datasets over DSC, respectively. From Figure 8, we can easily find out that the POIs' indexes generated by SHC^∗^ and DSC are more difficult to attack, because the estimated datasets are less similar to the original datasets. On the contrary, the estimated datasets reconstructed over the indexes built by SHC retain more details. It means that SHC^∗^ and DSC are more secure than SHC.

For further analysis, we calculate the* estimation distortion* of SHC, SHC^∗^, and DSC, and Table 3 shows the* estimation distortion* of SHC, SHC^∗^, and DSC for all datasets.

Because the* estimation distortion* represents the average error between the original and estimated datasets, larger metric value means more secure method. In Table 3, we can see that the* estimation distortion* of SHC is smaller than that of SHC^∗^ and DSC, and the* estimation distortion* of DSC is slightly smaller than that of SHC^∗^. It means that SHC^∗^ and DSC are more secure than SHC. As the clustering and distance-preserving performance of SHC is superior, the estimated datasets are more close to the original datasets, and the* estimation distortion* value is smaller. SHC^∗^ and DSC compress the* null value segments*, the distance-preserving property is somewhat violated, and malicious SP cannot speculate the location distribution of the original datasets by simply analyzing the POIs indexes, so SHC^∗^ and DSC are more secure.

## 6. Conclusion

In this paper, we propose SHC^∗^ and DSC for the index generation of outsourced spatial data and propose the* indistinguishability* for measuring the privacy disclosure risk of spatial transformation methods. The attack model is also defined, in the experiments, the estimated datasets are visualized for explicitly studying, and the* estimation distortion* shows that SHC^∗^ and DSC are more secure than SHC. The proposed methods can partially violate the distance-preserving property of SHC, so as to achieve better security. The index generation time shows that DSC is more efficient than SHC and SHC^∗^, so DSC achieves good security and efficiency performance. In the future, we will further conduct the security analysis of more spatial transformation methods and propose more effective metrics for the quantification of security.

## Figures and Tables

**Figure 9:**
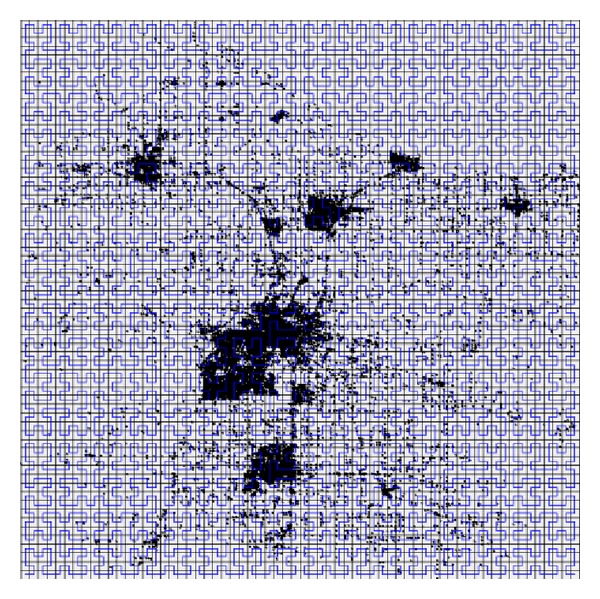
SHC over TG (*N* = 6).

**Figure 10:**
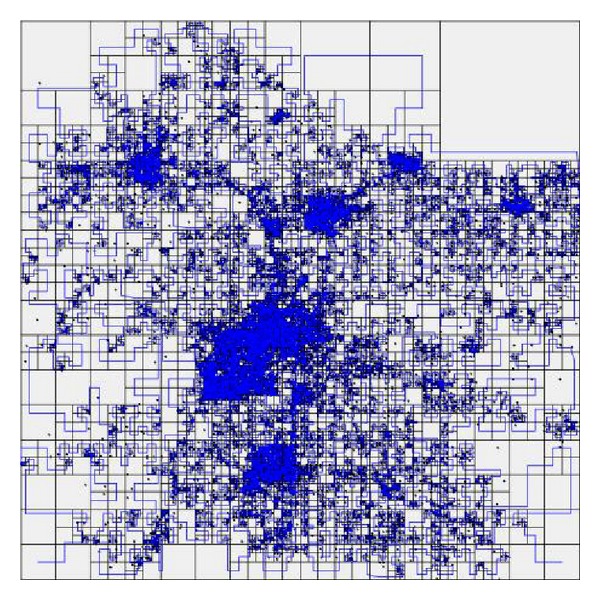
DSC over TG (*C* = 1).

**Algorithm 1:**
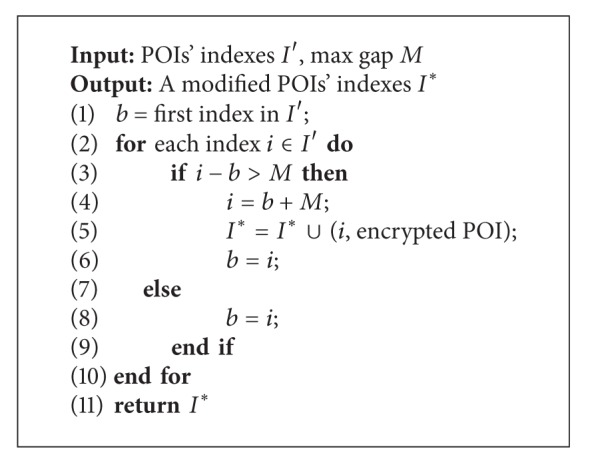
Index modification algorithm for SHC.

**Algorithm 2:**
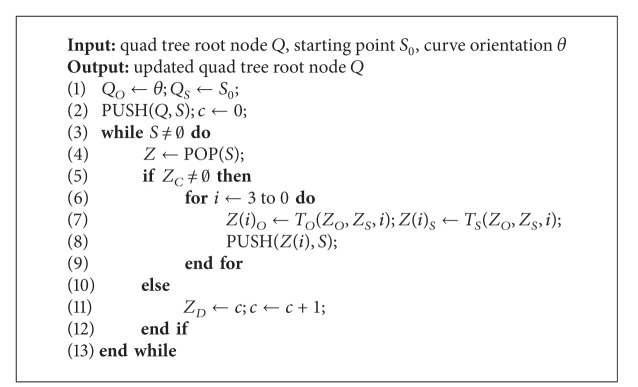
Index generation algorithm for DSC (IGD).

**Table 1:** Index generation time (ms).

Dataset	EDHO	BIA	SHC∗-E	SHC∗-B	IGD
NE	1115	863	1125	866	493
TG	154	111	156	113	65
SF	1585	1202	1610	1218	696
NA	1727	1315	1743	1326	998

**Table 2:** Indistinguishability.

Dataset	SHC	SHC∗	DSC
NE	10.6195	17.1513	16.7995
TG	10.1360	14.5252	13.9729
SF	9.1522	17.5486	17.2900
NA	9.7709	17.5766	17.3802

**Table 3:** Estimation distortion.

Dataset	SHC	SHC∗	DSC
NE	0.2547	0.3315	0.3236
TG	0.2835	0.4140	0.4079
SF	0.2103	0.3561	0.3418
NA	0.2473	0.3943	0.3706

## Appendix

Figures 9 and 10 show the different space partitions over dataset TG of SHC and DSC, respectively. The blue line represents the space filling curves. We can see that SHC partitions the spatial domain using the unified granularity, while DSC partitions the spatial domain according to the density of POIs. For convenience of observation, we set *N* = 6 for SHC and set *C* = 1 for DSC.
